# Tissue Engineering and Oral Rehabilitation in the Stomatognathic System

**DOI:** 10.1155/2017/4519568

**Published:** 2017-01-10

**Authors:** Tomasz Gedrange, Christiane Kunert-Keil, Friedhelm Heinemann, Marzena Dominiak

**Affiliations:** ^1^Department of Orthodontics, University Hospital TU Dresden, Fetscherstr. 74, 01307 Dresden, Germany; ^2^Department of Prosthodontics, Gerodontology and Biomaterials, Medical University of Greifswald, Rotgerberstr. 8, 17487 Greifswald, Germany; ^3^Department of Dental Surgery, Silesian Piast Medical University, 26 Krakowska St., 50-424 Wroclaw, Poland

The stomatognathic system is a functional unit characterized by several structures: skeletal components, dental arches, soft tissues, and the temporomandibular joint and masticatory muscles. These structures act in harmony to perform different functional tasks (to speak, to break food down into small pieces, and to swallow). On the other hand, the various components of the stomatognathic system influence each other. The restoration of function of the various components of the stomatognathic system is a major focus of research from different disciplines. For that reason stomatognathic diseases are treated by dentists and maxillofacial surgeons as well as otorhinolaryngologists. Furthermore, the preservation of the masticatory muscles is nowadays also at the forefront of scientists.

The current special issue summarizes five articles of different research aspects on the highly topical field of tissue engineering and oral rehabilitation in head and neck area. The issue presents a wide range of findings from basic and clinical research with interdisciplinary contributors from clinics, specialist doctors, and biologists.

The preservation of the bone with bone substitute materials as well as autologous bone plays an important role. Although these topics are explored for some time, there are always new aspects and improvements. T. Gredes et al., in Germany, in “Bone Regeneration after Treatment with Covering Materials Composed of Flax Fibers and Biodegradable Plastics: A Histological Study in Rats” have focused on bone regeneration using green biocomposites. These authors could demonstrate that composites from unmodified and transgenic modified flax fibers can be used as bone covering materials. The biocomposites prevent the growth of connective tissue into the bone defect.

Among other qualities of the bone, augmentation and surgical procedures play also an important role in the insertion of implants. Oral implantology is one of the fastest growing fields in oral rehabilitation. In implantology, minimally invasive surgical techniques are increasingly used, including piezosurgery. This is an ultrasound-assisted technique. During the operative preparation of the implantation bed, there is very often a strong heat development which can lead to thermal damage of the surrounding tissue. J. Matys et al. in Poland dealt with this topic* “*Assessment of Temperature Rise and Time of Alveolar Ridge Splitting by means of Er:YAG Laser, Piezosurgery, and Surgical Saw: An Ex Vivo Study.” They found no temperature rise on the bone over 10°C when using piezosurgery and Er:YAG laser as well as surgical saw and postulated that these surgical techniques are useful and safe for ridge splitting.

It is well known that a lot of factors can influence the osseointegration of dental implants and can cause dental implants failure. The manuscript by M. Prados-Privado et al. in Spain “Long-Term Fatigue and Its Probability of Failure Applied to Dental Implants” highlighted different fatigue analysis based on a cumulative damage model and probabilistic finite elements. With these analyses the authors found worst behavior of cylindrical implants compared to conical implants.

Furthermore, the function and composition of orofacial muscles are important for the maintenance of oral function. Patients with muscle diseases may suffer severe malocclusions, feeding difficulties, and weight loss, because of progressively impairing orofacial function. Furthermore it is known that the loss of teeth is followed by functional loss of orofacial muscles. It is becoming evident that more data about the muscle regeneration process are needed to develop strategies for improving life quality of the patients. The article by U. U. Botzenhart et al. from Germany “Influence of Botulinumtoxin A on the Expression of Adult MyHC Isoforms in the Masticatory Muscles in Dystrophin-Deficient Mice” fits very well with this topic. The authors describe that the neurotoxin botulinum toxin A causes muscular atrophy with signs of dystrophic phenotype in healthy mice, while mice with inherited muscle weakness do not show any changes in the MyHC isoform expression.

Many metabolic or hormonal diseases can influence both bone metabolism and muscle function. This is known, among other things, for hypothyroidism, which can lead to diminished length growth of the bones and myopathy. The work is complemented by a work by T. Świdziński et al. in Poland “Hypothyroidism Affects Olfactory Evoked Potentials.” These authors were able to demonstrate that the sense of smell is also strongly impaired in patients with clinical hypothyroidism, whereas patients with subclinical hypothyroidism do not show any swelling difficulties compared to healthy patients.

In conclusion, this special issue summarized various aspects of regeneration from different parts of the stomatognathic system. Both the implantology and the large area of bone substitute materials are still propagated in the focus of science.

*Tomasz Gedrange* *Christiane Kunert-Keil* *Friedhelm Heinemann* *Marzena Dominiak*

